# Level of adherence to physical activity recommendations among adults with type 2 diabetes in Qatar and associated factors: a cross-sectional study

**DOI:** 10.1186/s12889-025-22816-y

**Published:** 2025-04-26

**Authors:** Linda Boutefnouchet, Bushra Hoque, Layan Sukik, Mhd Osama Rahhal, Mohamed Elhadary, Ahmad Hamdan, Omar Altrmanini, Mohannad Natheef Abuhaweeleh, Hiba Bawadi, Mujahed Shraim

**Affiliations:** 1 Department of Human Nutrition, College of Health Sciences, QU Health, Qatar University, Doha, Qatar; 2 Department of Public Health, College of Health Sciences, QU Health, Qatar University, Doha, Qatar; 3 Infectious Disease Epidemiology Group, Weill Cornell Medicine-Qatar, Cornell University, Doha, Qatar; 4 World Health Organization Collaborating Centre for Disease Epidemiology Analytics on HIV/AIDS, Sexually Transmitted Infections, and Viral Hepatitis, Weill Cornell Medicine–Qatar, Cornell University, Qatar Foundation, Education City, Doha, Qatar; 5 College of Medicine, QU Health, Qatar University, Doha, Qatar

**Keywords:** Adherence, Physical activity recommendations, Adults, Type 2 diabetes, Cross-sectional study

## Abstract

**Background:**

Regular physical activity (PA) has beneficial health effects in controlling and managing diabetes. Identifying key factors associated with poor adherence to PA recommendations among patients with type 2 diabetes (T2D) has significant implications for future targeted interventions aimed at improving adherence and health outcomes in this population. The present study aims to determine the level of adherence to PA recommendations and the associated factors among adults with T2D in Qatar.

**Methods:**

This was a cross-sectional study in which secondary data collected from the Qatar Biobank were used. This was a population-based study including Qatari nationals and long-term residents. The amount of time spent performing moderate- and vigorous-intensity aerobic PA per week was self-reported. Adherence to PA recommendations was defined according to the WHO guidelines for adults and older adults (aged 18 years and older) with chronic conditions.

**Results:**

The study included 2,386 adults with T2D aged 18 years and older. Nine out of ten individuals failed to meet the PA recommendations, and 86.2% did not participate in any moderate- or vigorous-intensity aerobic PA. The main factors associated with not adhering to PA recommendations were older age, female sex, lower educational attainment, lower monthly income, treatment with tablets or tablets plus insulin compared with diet alone, sleeping less than 5 hours per day, and extended use of screen-based devices during weekends.

**Conclusions:**

Most adults with type 2 diabetes in Qatar do not meet physical activity recommendations, with lower adherence among older adults, females, those with high screen time, specific treatments, and lower education or income. Diabetes management and education should include targeted interventions to address barriers, while public health efforts should reduce sedentary behavior and improve access to physical activity for disadvantaged groups.

**Supplementary Information:**

The online version contains supplementary material available at 10.1186/s12889-025-22816-y.

## Background

Diabetes is a long-term metabolic condition characterized by high blood sugar levels and insulin resistance, and it poses a global public health problem [1]. In 2021, the International Diabetes Federation (IDF) estimated that 537 million people aged 20–79 years worldwide were living with diabetes, representing 10.5% of the global adult population; this percentage is predicted to increase to 11.3% (643 million) by 2030 and 12.2% (783 million) by 2045 [1]. The Middle East and North Africa had the highest comparative diabetes prevalence of 18.1% in 2021, which is expected to increase to 20.4% by 2045 [1]. Type 2 diabetes (T2D) accounts for approximately 90% of 537 million cases of diabetes worldwide [2]. Specifically, in Qatar, there has been an increasing trend in the total number of people with diabetes since the early 2000s [1]. In 2021, the number of people with diabetes in Qatar was reported to be 349,900, representing 16.4% of the total population, with projections indicating increases to 556,100 by 2030 and 795,500 by 2045 [1].

The multidisciplinary approach to T2D management involves an integrated plan of both medication treatment and lifestyle modification. Pharmacological therapy includes insulin treatment and oral hypoglycemic drugs such as metformin and sulfonylureas, whereas lifestyle modification includes a combination of a well-balanced diet that regulates carbohydrate intake and increased physical activity (PA) [3]. PA in the form of aerobic and resistance training contributes to improved health and function in different metabolic tissues and organs, including skeletal muscles, liver, adipose tissue, and pancreas, which leads to increased insulin action and glucose metabolism [4]. Research evidence has demonstrated that physical exercise confers beneficial effects that aid in the management of diabetes, such as increased energy expenditure and increased glucose uptake by skeletal muscles [5]. Moreover, continuous and regular exercise is associated with sustained improvements in insulin sensitivity, glucose disposal, and dyslipidemia while also reducing visceral adiposity and inflammation [5]. In accordance with the American Diabetes Association guidelines, engaging in moderate to vigorous aerobic exercise for at least 150 min/week for 3–7 days/week with no more than 2 consecutive days without physical exercise is recommended for adults with T2D [6]. Additionally, moderate to vigorous resistance exercise should include 8–10 repetitions and 1–3 sets per exercise, performed on at least 2 nonconsecutive days per week [6].

Despite compelling evidence supporting the benefits of regular PA in managing diabetes, the literature reveals wide global variations in adherence to PA recommendations among patients with T2D and in the factors associated with adherence. Research from several countries indicates that 25–79% of patients with T2D do not meet the minimum recommended PA levels based on self-reported measures [7–13]. These variations may be attributed to individual and regional factors, including age, gender, ethnicity, education, income, smoking, health status, family support, culture, and urbanization [8–12, 14–16]. They may also reflect regional resources constraints, challenges in implementing effective health promotion strategies, and limited awareness of the benefits of PA [17].

Despite the increasing burden of T2D in Qatar [18], no previous studies have examined factors associated with adherence to PA recommendations among patients with T2D. Identifying key factors associated with poor adherence to PA recommendations among patients with T2D has significant implications for future targeted interventions aimed at improving adherence and health outcomes in this population. Therefore, this study aims to determine the level of adherence to PA recommendations and the associated factors among adults with T2D in Qatar.

## Methods

### Study design, setting and population

The present study was reported according to the STROBE statement for reporting cross-sectional studies [19]; see supplemental file 1 for the checklist. This cross-sectional study utilized data collected by the Qatar Biobank (QBB) from adults with T2D. The QBB is a population-based study that includes Qatari nationals and long-term residents (those who have lived in Qatar for 15 years or more) aged 18 and older [20].

### Data collection

Since 2012, the QBB has been collecting intensive baseline data, including physical, clinical, and behavioral characteristics, from its study population, which reached 38,213 individuals in 2023 [21]. Full details about the variable measurements, data collection methods and instruments used by the QBB are published elsewhere [20]. In brief, through personal interviews with a nurse and self-administered questionnaires, the QBB gathered data on occupation, socioeconomic status, general health, current health conditions, family medical history, medication use, dietary habits, PA, and other lifestyle factors. The Institutional Review Board of Hamad Medical Corporation Ethics Committee authorized the research protocols of the QBB (Ex-2023-QF-QBB-RES-ACC-00172-0246). All participants provided written informed consent prior to enrolling in the QBB population-based study. The current study was approved by the QBB IRB for deidentified secondary data analysis (QF-QBB-RES-ACC-0169).

### Outcome variable

The time spent on moderate- and vigorous-intensity aerobic PA was evaluated via the International Physical Activity Questionnaire (IPAQ) [22], which was administered as part of the QBB study. The WHO recommends that all adults, including older adults with chronic conditions such as T2D, should aim for 150–300 min of moderate-intensity aerobic PA per week, 75–150 min of vigorous-intensity aerobic PA per week, or an equivalent combination of moderate- and vigorous-intensity aerobic PA per week [23]. Adherence to these guidelines for participants was determined based on their responses to the following two statements, which are part of the IPAQ measuring time spent on moderate and vigorous PA in the past 7 days: (a) Please indicate the total number of days per week and average time per day (hours and minutes) you engage in moderate physical activities (e.g., swimming or bicycling at a regular pace, yoga, doubles tennis, gym); (b) Please indicate the total number of days per week and average time per day (hours and minutes) you engage in vigorous physical activities (e.g., running, fast bicycling, fast swimming, aerobics, weight lifting, singles tennis, football). The outcome variable was adherence status to PA recommendations (adherent, not adherent) according to the WHO guidelines on PA for adults and older adults (aged 18 years and older) with chronic conditions [23].

### Predictor variables

The predictors included factors previously reported to be associated with adherence to PA among individuals with diabetes [8–11, 24, 25] and were collected via the QBB [20]. The predictors included age (years); sex (female, male); smoking status (no, yes, ex-smoker); monthly income reported in Qatari Riyal (< 10,000, 10,000–20,000, 20,001–50,000, > 50,000); and employment status (employed, retired, other). The level of education was classified as “low” (primary or secondary school), “medium” (technical or professional school), or “high” (university or postgraduate degree). BMI (kg/m222) was classified into the following categories according to the WHO BMI cutoff points: underweight (< 18.5), normal (18.5–24.9), overweight (25–29.9), and obese (≥ 30) [26]. Other predictors included diabetes duration (≤ 10 years, > 10 years); treatment type for diabetes (diet, tablets, insulin, tablets, and insulin); and number of hours of sleep per day (< 5, 5-<7, 7-<8, ≥ 8), including naps in a typical week during the previous year. In addition, time spent watching screen-based devices during weekends and weekdays was collected by asking the participants the following questions: “Please tell us about the time you have spent sitting per day during a typical week watching TV, DVDs, mobile phone, iPad etc.”; “Please tell us about the time you have spent sitting per day during weekends: watching TV, DVDs, mobile phone, iPad etc.”; “Please tell us about the time spent sitting per day using computer”. The answer options were as follows: none, less than one hour per day, between 1 h and less than 2 h per day, between two and less than four hours per day, and more than 4 h per day [20].

### Statistical analysis

The characteristics of the participants were described using the mean and standard deviation (SD) for age, as it was a normally distributed continuous variable, whereas frequencies and percentages were used for categorical variables. Bivariable and multivariable logistic regression, with a two-tailed significance level (alpha) of 0.05, were used to investigate the associations between the included predictors and adherence to PA recommendations. The odds ratio (OR) and 95% confidence interval (CI) were used as measures of association. Data analysis was performed using STATA version 18 [27].

## Results

### Subject characteristics

A total of 2,386 adults aged 18 years and older with T2D were included in the study. Table 1 presents the characteristics of the subjects. The subjects had a mean age of 51.6 years (SD = 11.8), and 59.4% of them were females. Among the participants, 60.8% were obese, 30.6% were overweight, 40.1% had a low education level, 51.5% had a monthly income higher than 20,000 in Qatari Riyal, and 18.6% were retired. Among the study participants, 12.1% were smokers, 65.1% reported sleeping for less than 7 hours per day, 21.0% spent more than four hours using screen-based devices on weekdays, and 19.9% did so on weekends. Among the subjects, 37.0% had T2D for more than 10 years, 19.9% were treated with diet alone, and 54.9% were treated with tablets (Table 1).

**Table 1:** Characteristics of participants (*n* = 2386)

Variable	Frequency (%)^c^
**Age (Years)** ^**a**^	51.6 (11.8)
**Sex**	
Female Male	1418 (59.4) 968 (40.6)
**BMI category** ^**b**^	
Normal Overweight Obese	204 (8.6) 731 (30.6) 1451 (60.8)
**Education level**	
Low Medium High	957 (40.1) 649 (27.2) 780 (32.7)
**Employment status**	
Employed Retired Other	1144 (48.0) 443 (18.6) 799 (33.5)
**Monthly income (Qatari Riyal)**	
< 10,000 10,000–20,000 20,001–50,000 > 50,000	646 (27.1) 511 (21.4) 699 (29.3) 530 (22.2)
**Smoking status**	
No Yes Ex-smoker	1770 (74.2) 289 (12.1) 327 (13.7)
**Number of hours spent sitting per day watching TV**,** DVD**,** tablet**,** and phone during weekdays**	
Less than 1 h 1 to 2 h 2 to 4 h More than 4 h	623 (26.1) 661 (27.7) 600 (25.2) 502 (21.0)
**Number of hours spent sitting per day watching TV**,** DVD**,** tablet**,** and phone during weekends**	
Less than 1 h 1 to 2 h 2 to 4 h More than 4 h	782 (32.8) 592 (24.8) 537 (22.5) 475 (19.9)
**Number of sleep hours per day**	
< 5 5-<7 7-<8 ≥ 8	395 (16.6) 1158 (48.5) 603 (25.3) 230 (9.6)
**Diabetes treatment**	
Diet Tablets Insulin Tablets and Insulin	474 (19.9) 1310 (54.9) 248 (10.4) 354 (14.8)
**Diabetes duration**	
≤ 10 years > 10 years Do not know	930 (39.0) 883 (37.0) 573 (24.0)

BMI denotes Body Mass Index; ^a^ mean (SD); ^b^ two individuals had a BMI < 18.5 and were included in the normal range category; ^c^ some reported percentages may not add up to 100% due to rounding

### Level of adherence to PA recommendations

Among the study participants, only 10.5% met the PA recommendations, 3.3% participated in moderate- and/or vigorous-intensity aerobic PA per week below the recommendations, whereas 86.2% did not engage in any moderate- or vigorous-intensity aerobic PA per week.

### Factors associated with adherence to PA recommendations

**Fig. 1:**
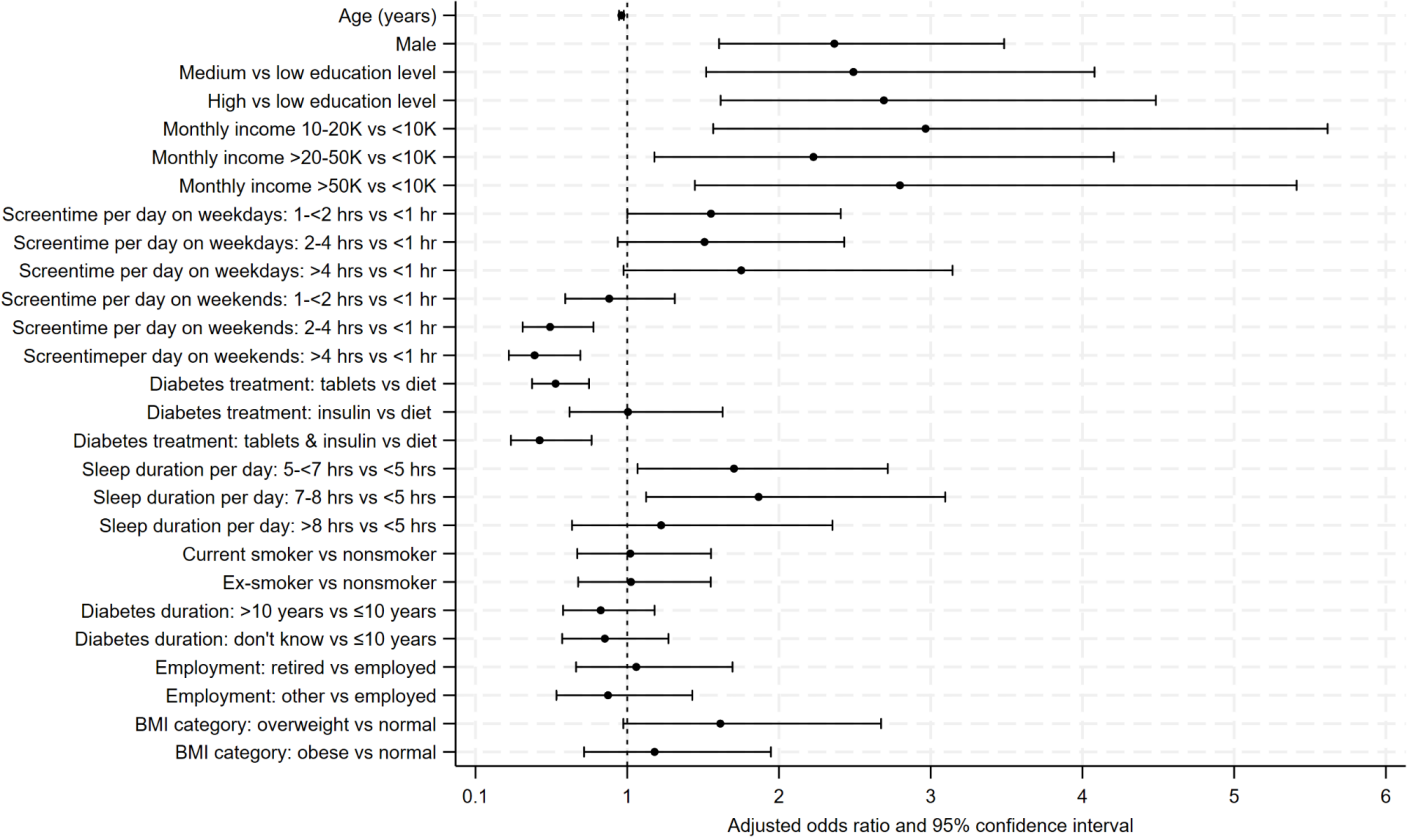
Adjusted odds ratios and 95% confidence intervals for the associations between individual characteristics and adherence to physical activity recommendations

Supplemental Table 1 presents crude and adjusted associations between predictor variables and adherence to PA recommendations, and Fig. 1 depicts only the adjusted associations. In the multivariable analysis, age, hours spent sitting and watching screen-based devices during weekends, and type of diabetes treatment were inversely associated with PA adherence. An increase in age by one year was associated with lower odds of adhering to PA recommendations (adjusted OR 0.96; 95% CI 0.95, 0.98). Compared with those spending less than one hour, those spending 2 to 4 hours and more than 4 hours sitting and watching screen-based devices had significantly lower odds of adhering to PA recommendations (aOR 0.49; 95% CI 0.32, 0.77 and aOR 0.38; 95% CI 0.22, 0.68, respectively). However, this association was not statistically significant for subjects spending 1–2 hours (aOR 0.85; 95% CI 0.57, 1.26). Compared with those treated with diet alone, those treated with tablets and tablets plus insulin had significantly lower odds of adhering to PA recommendations (aORs of 0.52; 95% CIs of 0.37, 0.73 and 0.40; 95% CIs of 0.22, 0.70, respectively). However, no statistically significant difference was observed for subjects treated with insulin alone compared with those fed a diet alone (aOR 0.94; 95% CI 0.60, 1.47).

Male sex, higher education level, higher monthly income, and increased sleep hours were associated with higher odds of adhering to PA recommendations. Compared with females, males had greater odds of adhering to PA recommendations (aOR 2.55; 95% CI 1.87, 3.46). Higher education levels were associated with increased odds of adhering to PA recommendations: “medium” education (aOR 2.61; 95% CI 1.61, 4.23) and “high” education (aOR 2.95; 95% CI 1.82, 4.77) compared with “low education”. Higher income categories were also associated with higher odds of adherence to PA recommendations than a monthly income of less than 10,000 Qatari Riyals: 10,000 to 20,000 Qatari Riyals (aOR 3.05; 95% CI: 1.64, 5.71), 20,001 to 50,000 Qatari Riyals (aOR 2.35; 95% CI: 1.26, 4.36), and over 50,000 Qatari Riyals (aOR 2.94; 95% CI: 1.54, 5.63). Similarly, subjects reporting 5 to less than 7 hours and 7 to less than 8 hours of sleep per day had higher odds of adhering to PA recommendations (aOR 1.73; 95% CI 1.09, 2.75 and aOR 1.88; 95% CI 1.14, 3.12, respectively) than those sleeping less than 5 hours. However, this association was not statistically significant for those reporting 8 hours or more of sleep (aOR 1.25; 95% CI 0.65, 2.39). Compared with those who spent less than one hour, those who spent 1–2 hours sitting and watching screen-based devices during weekdays had 1.57 times greater odds of adhering to PA recommendations (95% CI 1.01, 2.42). However, this association did not reach statistical significance for those spending 2 to 4 hours (aOR 1.48; 95% CI 0.92, 2.38) or more than 4 hours (aOR 1.74; 95% CI 0.98, 3.11).

Conversely, subjects who spent 2–4 hours and more than 4 hours sitting and watching screen-based devices during weekends had lower odds of adhering to PA recommendations than did those who spent less than one hour (aOR 0.49; 95% CI 0.31, 0.77 and aOR 0.38; 95% CI 0.22, 0.68, respectively). However, this association was not statistically significant for those spending 1–2 hours (aOR 0.85; 95% CI 0.57, 1.26). Finally, no statistically significant associations were observed between BMI, smoking status, employment status, or duration of T2D and adherence to PA recommendations in the multivariable analysis (Fig. 1 and Supplemental Table 1).

## Discussion

This study is the first to assess the level of adherence to PA recommendations and associated factors among adults with T2D in Qatar. Our study found that the majority of patients with T2D did not meet the recommended PA levels. Older age, increased weekend screen time, and diabetes treatment modality were negatively associated with adherence to PA recommendations. In contrast, male sex, higher education, higher income, and adequate sleep were positively associated with adherence to PA recommendations. Our findings showed that approximately 9 out of 10 adults with T2D do not meet the PA recommendations. This proportion is significantly greater than those reported in previous studies from Saudi Arabia (56% [8] and 70% [9]), Nepal (46% [7] and 79% [10]), Ethiopia (64%) [11], and the U.S. (75%) [12]. The exact reason for this is unclear, but it may be influenced by various interacting factors, such as hot weather limiting outdoor PA and cultural norms and religious beliefs restricting female participation in regular PA outside their homes [28, 29].

Our finding that increasing age is associated with lower odds of adhering to PA recommendations is consistent with the findings of previous studies among adults with T2D [8, 9, 11]. One plausible explanation is that increasing age is associated with various factors reported as barriers to participating in PA, such as comorbid conditions (e.g., heart disease, arthritis, physical disability), tiredness, pain, negative emotions, diabetic foot, and fear of injury and hypoglycemia [28]. In line with other studies [9, 11], females had lower odds of adhering to PA recommendations than males did. This disparity may be attributed to factors such as a lack of time due to family and household responsibilities, cultural norms and religious beliefs that limit female participation in regular PA outside their homes, and the accessibility and availability of recreational facilities for women [29]. For example, in Muslim communities, the taboo against females going out in public places unless accompanied by a male family member has been reported as a barrier to PA participation among women [30, 31]. Our study indicated that individuals with lower educational attainment were at lower odds of adhering to AP recommendations, which is consistent with findings from previous research [9, 11]. This may be explained by lower levels of self-efficacy in managing diabetes, a factor commonly associated with low educational level [32, 33]. Our findings, which are consistent with those of previous studies [9, 10, 34], revealed that lower monthly income was associated with lower odds of adhering to PA recommendations. This may be due to financial constraints and limited access to recreational and PA facilities, which has been identified as a barrier to regular PA participation among individuals with T2D [28].

The present study revealed that individuals who reported sleeping less than 5 hours per day were at lower odds of adhering to PA recommendations than individuals who reported sleeping for longer durations. This finding aligns with longitudinal and experimental studies showing that poor and inadequate sleep durations are associated with reduced daytime PA due to fatigue and sleepiness [35, 36]. In addition, our results showed that individuals who spent more hours per day setting and using screen-based devices had lower odds of adhering to PA recommendations. This is consistent with the findings of systematic reviews demonstrating that individuals with sedentary behavior are less likely to engage in regular PA [28, 29]. Our findings regarding diabetes treatment modalities revealed that subjects treated with tablets and tablets plus insulin presented significantly lower odds of adhering to PA recommendations than did those managed with diet alone. The mechanisms underlying this are not clear, but fear of exercise-induced hypoglycemia, which has been reported as a barrier to engaging in regular PA among individuals with T2D [28], could explain part of this association.

In line with the findings of a systematic review [28], we found no statistically significant associations between smoking, employment, or duration since the onset of T2D and adherence to PA recommendations, suggesting that these factors may not be significant barriers to PA participation among individuals with T2D. Our study revealed no statistically significant association between obesity and adherence to PA recommendations, which contrasts with the findings of a large U.S. study [34]. In the study by Morrato and colleagues, 75% of the participants did not adhere to the PA recommendations, with 36.4% having a normal weight, 36.0% being overweight, and 27.6% being obese. In our study, 89.5% of the participants did not adhere, with 8.6% having a normal weight, 30.6% being overweight, and 60.8% being obese [34]. The high prevalence of both obesity and nonadherence to PA recommendations in our study, along with differences in BMI distribution, may partially explain the discrepancy.

The effective dissemination of PA guidelines through structured patient education, public health campaigns, and culturally tailored community-based health promotion initiatives is a critical prerequisite for promoting adherence to PA recommendations. These efforts are foundational, providing individuals with clear, consistent, and accessible information that raises awareness, enhances knowledge, and facilitates behavior change, thereby supporting the integration of PA into daily routines. Culturally tailored community-based interventions, in particular, are essential, as they offer accessible PA opportunities within local contexts and foster supportive environments that mitigate key barriers to sustained participation in PA. Our finding that 9 out of 10 individuals with T2D do not meet the recommended PA guidelines, which vary according to demographic, behavioral, and clinical factors, has significant public health implications. This study underscores the need for comprehensive strategies to promote PA, including regular individualized counseling and support aimed at enhancing motivation and self-efficacy. These interventions should be culturally and individually tailored to overcome barriers that impede regular PA engagement. Additionally, future qualitative and longitudinal research is essential to better understand the mechanisms underlying low PA engagement. Such insights are crucial for improving the development and delivery of accessible PA opportunities that resonate with diverse populations with T2D in Qatar, ultimately enhancing adherence to PA recommendations.

A primary strength of our study is the utilization of comprehensive data encompassing key demographic, socioeconomic, behavioral, and clinical characteristics from a large national sample of patients with T2D. Our study also has several limitations. *First*, this study was cross-sectional; thus, the temporal sequence of some associations and potential changes in PA participation over time remain unclear. *Second*, our study lacked data on several factors previously associated with PA participation, such as knowledge or awareness of the benefits of PA, physical and mental health conditions, physical pain and disability, fear of injury or hypoglycemia, family and social support, cultural barriers to PA participation, and access to exercise facilities [28]. *Third*, the amount of time spent per week performing moderate and/or vigorous-intensity aerobic PA was self-reported, which may introduce recall bias and potentially misclassify adherence to PA recommendations. However, we do not anticipate that this significantly impacts PA adherence status, given that 86.2% of individuals did not engage in any form of moderate- or vigorous-intensity aerobic PA.

## Conclusions

The vast majority of adult patients with type 2 diabetes in Qatar do not meet the recommended physical activity levels, with lower adherence observed among older adults, females, individuals with increased weekend screen time, specific diabetes treatments, and those with lower education, income, or inadequate sleep. To improve adherence to physical activity recommendations, diabetes management and education should incorporate targeted, tailored interventions that address barriers and provide enhanced support. Public health efforts should focus on reducing sedentary behaviors, particularly excessive screen time, while policies should improve access to physical activity opportunities for individuals with lower income and education.

## Electronic supplementary material

Below is the link to the electronic supplementary material.

Supplementary Material 1

Supplementary Material 2

## Data Availability

The data that support the findings of this study are available from Qatar Biobank but restrictions apply to the availability of these data, which were used under license for the current study, and so are not publicly available. Data are however available from the authors upon reasonable request and with permission of Qatar Biobank.

## Abbreviations

aOR: Adjusted odds ratio
BMI: Body mass index
CI: Confidence interval
IDF: International Diabetes Federation
OR: Odds ratio
P: Probability
PA: Physical activity
QBB: Qatar Biobank
Ref: Reference Category
SD: Standard deviation
STROBE: Strengthening the reporting of observational studies in epidemiology
T2D: Type 2 diabetes
WHO: World Health Organization
