# Supplementary material for: Level of adherence to physical activity recommendations among adults with type 2 diabetes in Qatar and associated factors: a cross-sectional study
Source: BMC Public Health. 2025 Apr 26;25:1553. doi: 10.1186/s12889-025-22816-y (PMC12032733; doi:10.1186/s12889-025-22816-y)
Supplement: Supplementary file 1 — Supplementary Material 1 [file 12889_2025_22816_MOESM1_ESM.docx]

**Supplemental Table 1** Crude and adjusted associations between individual characteristics and adherence to physical activity recommendations (n= 2386)

| **Variable** | **Adherence to PA recommendations** | | **Crude association** | | | **Adjusted association** | | |
| --- | --- | --- | --- | --- | --- | --- | --- | --- |
|  | **Yes (n=251)**  **N (%)** | **No (n=2,135)**  **N (%)** | **OR (95% CI)** | **SE** | **p** | **OR (95% CI)** | **SE** | **p** |
| **Age** (mean + SD) | 44.6 (11.3) | 52.5 (11.6) | 0.95 (0.94-0.96) | 0.01 | <0.001 | 0.96 (0.95, 0.98) | 0.01 | <0.001 |
| **Sex**  Female  Male | 90 (6.4)  161 (16.6) | 1328 (93.7)  807 (83.4) | Ref  2.94 (2.24-3.87) | 0.41 | <0.001 | Ref  2.55 (1.87, 3.46) | 0.40 | <0.001 |
| **BMI category**  Normal  Overweight  Obese | 24 (11.8)  110 (15.1)  117 (8.1) | 180 (88.2)  621 (85.0)  1334 (91.9) | Ref  1.33 (0.83-2.13)  0.66 (0.41-1.05) | 0.32  0.16 | 0.238  0.078 | Ref  1.63 (0.98, 2.69)  1.15 (0.70, 1.90) | 0.42  0.29 | 0.059  0.582 |
| **Education level**  Low  Medium  High | 27 (2.8)  99 (15.3)  125 (16.0) | 930 (97.2)  550 (84.8)  655 (84.0) | Ref  6.20 (4.00-9.61)  6.57 (4.29-10.08) | 1.39  1.43 | <0.001  <0.001 | Ref  2.61 (1.61, 4.23)  2.95 (1.82, 4.77) | 0.64  0.72 | <0.001  <0.001 |
| **Employment status**  Employed  Retired  Other | 185 (16.2)  34 (7.7)  32 (4.0) | 959 (83.8)  409 (92.3)  767 (96.0) | Ref  0.43 (0.29-0.63)  0.22 (0.15-0.32) | 0.08  0.04 | <0.001  <0.001 | Ref  1.03 (0.65, 1.65)  0.88 (0.54, 1.43) | 0.25  0.22 | 0.900  0.600 |
| **Monthly income (Qatari Riyal)**  Less than 10,000  Between 10,000 and 20,000  Between 20,001 and 50,000  More than 50,000 | 14 (2.2)  61 (11.9)  103 (14.7)  73 (13.8) | 632 (97.8)  450 (88.1)  596 (85.3)  457 (86.2) | Ref  6.12 (3.38-11.08)  7.80 (4.41-13.79)  7.21 (4.02-12.94) | 1.85  2.27  2.15 | <0.001  <0.001  <0.001 | Ref  3.05 (1.64, 5.71)  2.35 (1.26, 4.36)  2.94 (1.54, 5.63) | 0.97  0.74  0.97 | <0.001  0.007  0.001 |
| **Smoking status**  No  Yes  Ex-Smoker | 146 (8.3)  54 (18.7)  51 (15.6) | 1624 (91.8)  235 (81.3)  276 (84.4) | Ref  2.56 (1.82-3.59)  2.06 (1.46-2.90) | 0.44  0.36 | <0.001  <0.001 | Ref  1.02 (0.67, 1.54)  1.01 (0.67, 1.53) | 0.22  0.21 | 0.939  0.958 |
| **Number of hours spent sitting per day watching TV, DVD, tablet, and phone during weekdays**  Less than 1 hour  1 to 2 hours  2 to 4 hours  More than 4 hours | 53 (8.5)  81 (12.3)  71 (11.8)  46 (9.2) | 570 (91.5)  580 (87.8)  529 (88.2)  456 (90.8) | Ref  1.50 (1.04-2.16)  1.44 (0.99-2.10)  1.08 (0.72-1.64) | 0.28  0.28  0.23 | 0.029  0.055  0.699 | Ref  1.57 (1.01, 2.42)  1.48 (0.92, 2.38)  1.74 (0.98, 3.11) | 0.35  0.36  0.52 | 0.043  0.103  0.061 |
| **Number of hours spent sitting per day watching TV, DVD, tablet, and phone during weekends**  Less than 1 hour  1 to 2 hours  2 to 4 hours  More than 4 hours | 85 (10.9)  76 (12.8)  52 (9.7)  38 (8.0) | 697 (89.1)  516 (87.2)  485 (90.3)  437 (92.0) | Ref  1.21 (0.87-1.68)  0.88 (0.61-1.27)  0.71 (0.48-1.06) | 0.20  0.16  0.15 | 0.262  0.488  0.098 | Ref  0.85 (0.57, 1.26)  0.49 (0.31, 0.77)  0.38 (0.22, 0.68) | 0.17  0.11  0.11 | 0.425  0.002  0.001 |
| **Number of sleep hours per day**  Less than 5 hours  Between 5 and less than 7 hours  Between 7 and less than 8 hours  8 hours or more | 26 (6.6)  137 (11.8)  68 (11.3)  20 (8.7) | 369 (93.4)  1021 (88.2)  535 (88.7)  210 (91.3) | Ref  1.90 (1.23-2.94)  1.80 (1.13-2.89)  1.35 (0.74-2.48) | 0.42  0.43  0.42 | 0.004  0.014  0.331 | Ref  1.73 (1.09, 2.75)  1.88 (1.14, 3.12)  1.25 (0.65, 2.39) | 0.41  0.48  0.41 | 0.021  0.014  0.504 |
| **Diabetes treatment**  Diet  Tablets  Insulin  Tablets and Insulin | 90 (19.0)  104 (7.9)  40 (16.1)  17 (4.8) | 384 (81.0)  1206 (92.1)  208 (83.9)  337 (95.2) | Ref  0.37 (0.27-0.50)  0.82 (0.55-1.23)  0.22 (0.13-0.37) | 0.06  0.17  0.06 | <0.001  0.343  <0.001 | Ref  0.52 (0.37, 0.73)  0.94 (0.60, 1.47)  0.40 (0.22, 0.70) | 0.09  0.21  0.12 | <0.001  0.796  0.002 |
| **Diabetes duration**  10 years or less  More than 10 years  Do not know | 130 (14.0)  77 (8.7)  44 (7.7) | 800 (86.0)  806 (91.3)  529 (92.3) | Ref  0.59 (0.44-0.79)  0.51 (0.36-0.73) | 0.09  0.09 | <0.001  <0.001 | Ref  0.86 (0.61, 1.23)  0.87 (0.58, 1.29) | 0.16  0.18 | 0.421  0.482 |

PA denotes physical activity; Cl, confidence interval; OR, odds ratio; Ref, reference category; SD, standard deviation; SE, standard error; p, probability. Some percentages reported may not add up to 100% due to rounding.
